# Synergistic association of resveratrol and histone deacetylase inhibitors as treatment in amyotrophic lateral sclerosis

**DOI:** 10.3389/fphar.2022.1017364

**Published:** 2022-10-21

**Authors:** Edoardo Parrella, Vanessa Porrini, Ilaria Scambi, Michele M. Gennari, Cristina Gussago, Oluwamolakun Bankole, Marina Benarese, Raffaella Mariotti, Marina Pizzi

**Affiliations:** ^1^ Division of Pharmacology, Department of Molecular and Translational Medicine, University of Brescia, Brescia, Italy; ^2^ Section of Anatomy and Histology, Department of Neuroscience, Biomedicine and Movement Sciences, University of Verona, Verona, Italy

**Keywords:** amyotrophic lateral sclerosis (ALS), NF-κB/RelA, histone acetylation, resveratrol, histone deacetylase (HDAC) inhibitors, epigenetic drugs, sexual dimorphism

## Abstract

Amyotrophic lateral sclerosis (ALS) is a fatal neurodegenerative disease associated with motor neuron degeneration, progressive paralysis and finally death. Despite the research efforts, currently there is no cure for ALS. In recent years, multiple epigenetic mechanisms have been associated with neurodegenerative diseases. A pathological role for histone hypoacetylation and the abnormal NF-κB/RelA activation involving deacetylation of lysines, with the exclusion of lysine 310, has been established in ALS. Recent findings indicate that the pathological acetylation state of NF-κB/RelA and histone 3 (H3) occurring in the SOD1(G93A) murine model of ALS can be corrected by the synergistic combination of low doses of the AMP-activated kinase (AMPK)-sirtuin 1 pathway activator resveratrol and the histone deacetylase (HDAC) inhibitors MS-275 (entinostat) or valproate. The combination of the epigenetic drugs, by rescuing RelA and the H3 acetylation state, promotes a beneficial and sexually dimorphic effect on disease onset, survival and motor neurons degeneration. In this mini review, we discuss the potential of the epigenetic combination of resveratrol with HDAC inhibitors in the ALS treatment.

## Introduction

Amyotrophic lateral sclerosis (ALS) is a fatal neurodegenerative disease associated with motor neurons (MNs) degeneration, muscle weakness, fasciculations, muscle atrophy, swallowing and speech disabilities, paralysis and finally death (van Es et al., 2017). Death is usually caused by respiratory failure and occurs in 2–4 years after the onset (Huisman et al., 2011; Wittie et al., 2013; Oskarsson et al., 2018). ALS occurs with an incidence of 1–2 cases in 100,000 individuals per year, with about 90% of cases being sporadic and 10% characterized as familial (Oskarsson et al., 2018). More than 20 genes have been associated with familial ALS, of which the ones encoding for chromosome nine open reading frame 72 (C9orf72), superoxide dismutase 1 (SOD1), Fused in sarcoma (FUS), and TAR DNA-binding protein 43 (TDP-43), account for most of the cases (Chia et al., 2018).

However, despite decades of research, the mechanisms underlying ALS pathogenesis remain unclear. ALS appears as a multifactorial disease where several processes operate simultaneously. These include oxidative stress, glutamate excitotoxicity, mitochondrial dysfunction, inflammatory response, impairment of axonal transport, impaired protein homeostasis and transcriptional dysregulation (Giribaldi et al., 2013; Mejzini et al., 2019; Bendotti et al., 2020; De Marchi et al., 2021).

Epidemiological studies and clinical observations have shown evidence of sexual dimorphism in ALS (Vegeto et al., 2020). Men display higher risk of developing ALS, with a male/female ratio reported between 1 and 3, depending on geographic area and population considered (Manjaly et al., 2010). Although the overall survival is similar in both sexes, the disease appears earlier in men (Blasco et al., 2012). Moreover, the ALS phenotype is different in males and females, with a predominance of limb onset in men and bulbar onset in women (Blasco et al., 2012).

Currently, no cure is available for ALS and the molecules tested, alone or in combinations, in animal models and in patients did not lead to real improvements (Mitsumoto et al., 2014; Wobst et al., 2020). The only therapeutic drugs approved for ALS treatment are riluzole, a glutamate receptor antagonist approved in 1995, and edavarone, a free radical scavenger approved by the FDA in 2017 (Oskarsson et al., 2018; Yoshino 2019; Wobst et al., 2020). Riluzole has been shown in clinical trials to prolong median survival from 11.8 to 14.8 months, postponing the use of surrogate approaches, such as tracheotomy and mechanical ventilation (Lacomblez et al., 1996; Miller et al., 2012). Edavarone modestly slows the rate of disease progression and prolongs the tracheostomy-free survival in ALS patients (Writing Group and Edaravone (MCI-186) ALS 19 Study Group. 2017; Okada et al., 2018).

## The pathogenic role of anomalous acetylation of NF-κB/RelA and histones in ALS

In the central nervous system (CNS), the nuclear factor kappa-light-chain enhancer of activated B cells (NF-κB) transcription factors play a pivotal role in a number of physiological processes including neurogenesis (Zhang and Hu, 2012), neuritogenesis (Gutierrez and Davies, 2011), learning and memory (Kaltschmidt and Kaltschmidt, 2015). On the other hand, NF-κB dysregulation has been associated to neurodegenerative mechanisms occurring in pathological conditions such as stroke, epilepsy, Parkinson’s disease (PD) and Alzheimer’s disease (AD) (Srinivasan and Lahiri, 2015; Bellucci et al., 2020).

The NF-κB family is composed by five different members [p50/p105 (NF-κB1), p52/p100 (NF-κB2), p65 (RelA), c-Rel and RelB] that combine to form the transcriptionally active dimer (Gilmore and Wolenski 2012; Lanzillotta et al., 2015). Neurotoxic stimuli, including ischemia (Inta et al., 2006), glutamate (Pizzi et al., 2002), β-amyloid (Pizzi et al., 2005), or 1-methyl-4-phenylpyridinium (MPP+) (Ghosh et al., 2007; Sarnico et al., 2008) induce the activation of p50/RelA dimer and the transcription of pro-apoptotic factors, such as Bim and Noxa. The opposite effects exerted by the NF-κB dimer p50/RelA on neuron survival can rely on changes in the acetylation state of RelA within the nuclear p50/RelA (Lanzillotta et al., 2010; Sarnico et al., 2012). In particular, a specific pro-apoptotic acetylation profile of nuclear RelA, involving the general deacetylation of lysines but a site-specific acetylation at the lysine 310 [acRelA(K310)], occurs in lethal ischemia but not in protective preconditioning brain ischemia (Lanzillotta et al., 2010). The correction of RelA acetylation by pharmacological HDAC modulation (see below) reduced both the brain infarct volume and the neurological deficits (Lanzillotta et al., 2013; Faggi et al., 2018).

A pathogenic role for the activation of NF-κB/RelA has been suggested also for ALS.

Major genetic risk factors linked to ALS, including mutations in genes encoding for SOD1, OPTN, TBK1, TDP-43 and FUS, activate the NF-κB pathway (Källstig et al., 2021). NF-κB/RelA levels were elevated in mutant SOD1 MNs and astrocytes cellular model of ALS (Prell et al., 2014; Ikiz et al., 2015; Yin et al., 2018). Interestingly, Yin and colleagues reported that the MNs vulnerability to the conditioned medium obtained from mutant SOD1 astrocytes was dependent on the activation of the phosphorylated form of RelA, a modification known to promote RelA acetylation at the K310 residue (Yin et al., 2018). NF-κB/RelA was increased with disease progression in the SOD1(G93A) and TDP-43 mouse models of ALS (Swarup et al., 2011; Frakes et al., 2014). Neuron-specific expression of super-repressor form of the inhibitory kB proteins (IκB-SR) ameliorated behavioral and pathologic phenotypes in three mouse models of ALS carrying either human mutated TDP-43 or SOD1 transgenes (Dutta et al., 2020). Furthermore, NF-κB inhibition by administration of withaferin A alleviated disease symptoms in TDP-43 mice (Swarup et al., 2011) and extended lifespan of SOD1(G93A) and SOD1(G37R) mouse models of ALS (Frakes et al., 2014; Patel et al., 2015). In addition, levels of NF-κB/RelA were higher also in the spinal cord of ALS patients when compared with age-matched healthy subjects (Jiang et al., 2005; Swarup et al., 2011). Notably, as previously found in ischemic brain neurons (Lanzillotta et al., 2010; Lanzillotta et al., 2013), the pro-apoptotic acetylation profile acRelA(K310) was observed also in the lumbar spinal cord of SOD1(G93A) mice (Schiaffino et al., 2018; Bankole et al., 2022). These results suggest that NF-κB signaling may represent a unique therapeutic target for ALS disease. The beneficial effect of a therapeutic approach inhibiting NF-κB/RelA could be enormously enhanced by switching-off the p50/RelA pro-inflammatory/pro-apoptotic activity without modifying the dimer effects on neurogenesis, neuritogenesis and cell survival.

Histone acetylation at lysine residues is an important epigenetic mechanism regulating chromatin folding and the accessibility of transcription factors to their target genes (Eberharter and Becker 2002). Altered histone acetylation has been associated with reduced neuronal survival and pathological CNS conditions, including stroke, PD, AD and Huntington’s disease (Konsoula and Barile, 2012; Uzdensky and Demyanenko 2021). A growing body of evidence suggests a possible role for a dysregulation of epigenetic mechanisms, including histone acetylation, also in the occurrence and progression of ALS (Jimenez-Pacheco et al., 2017; Bennett et al., 2019; Zhang et al., 2022). For example, overexpression of FUS or TDP-43 in yeast ALS proteinopathy models resulted in histone hypo- and hyperacetylation, respectively, suggesting that each proteinopathy may correspond to a specific alteration of histone acetylation (Chen K et al., 2018). Moreover, histone acetylation was reduced in the spinal cord of SOD1(G93A) and Tg FUS^+/+^ mouse models of ALS (Schiaffino et al., 2018; Rossaert et al., 2019; Bankole et al., 2022). In light of these considerations, histone acetylation appears as a potential target for ALS treatment.

The acetylation state of NF-κB/RelA and histones results from the opposing activity of histones acetyltransferases (HATs) and histone deacetylases (HDACs).

Until now, eighteen mammalian HDACs have been characterized and grouped into four major classes according to their homology with yeast HDACs (Shukla and Tekwani, 2020). The Class I, II, and IV are known as classical HDACs and use zinc as cofactor. Class III HDACs, commonly known as sirtuins (SIRT 1–7), are NAD dependent and are involved in regulation of metabolism, stress and aging. Members of class I HDAC (HDAC 1, 2, 3 and 8) are most involved in the regulation of acetylation state of NF-κB/RelA (Chen and Greene, 2004). A body of evidence showed an unbalance of HATs and HDACs activity in patients as well as in preclinical models of ALS (Schmalbach and Petri, 2010; Lazo-Gómez et al., 2013; Jimenez-Pacheco et al., 2017; Shukla and Tekwani, 2020; Klingl et al., 2021). HDAC1 silencing or treatment with pan-HDAC inhibitors exert a protective role against wild-type or pathological mutant TDP-43 toxicity, suggesting TDP-43 acetylation as a new potential therapeutic target (Sanna et al., 2020). While data from expression analysis of HDAC isoforms in post-mortem brain and spinal cord tissue of ALS patients remain controversial (Janssen et al., 2010; Dios et al., 2019), recent findings suggest that increasing HDACs activity might exert a protective role in ALS. This is the case of the class IIa HDAC 4, whose expression in skeletal muscle of an ALS mouse model is responsible for compensatory reinnervation (Pigna et al., 2019).

## The anomalous acetylation of NF-κB/RelA and histones can be corrected by the association of resveratrol and HDAC inhibitors

We reported that the pathological acetylation profile of RelA and histones in brain ischemia can be corrected by the synergistic combination of low doses of the epigenetic drugs resveratrol and the HDAC inhibitor MS-275 (entinostat) (Lanzillotta et al., 2013).

Resveratrol is a polyphenol stilbene widely investigated for the prevention or treatment of different diseases thanks to its anti-aging, anti-inflammatory, anti-oxidant and anti-tumorigenic properties (Rauf et al., 2017; Parrella et al., 2020; Zhang et al., 2021). Among its multiple mechanisms of action, the molecule is able to activate the class III NAD + -dependent HDAC SIRT1 (Lee et al., 2019) and AMP-activated protein kinase (AMPK), a serine–threonine kinase acting as a key metabolic and stress sensor/effector (Ruderman et al., 2010).

The synthetic benzamide MS-275 has been shown to inhibit class I HDAC (HDAC 1–3) with excellent pharmacokinetic properties (Simonini et al., 2006; Khan et al., 2008). The molecule is in clinical trials for the treatment of different types of cancer (Wang et al., 2022).

The use of resveratrol and MS-275 promoted a synergistic neuroprotection in primary cortical neurons exposed to oxygen and glucose deprivation (OGD) and in mice subjected to transient middle cerebral artery occlusion (tMCAO) (Lanzillotta et al., 2013). Similarly, a single treatment with resveratrol and MS-275 reduced stroke-mediated brain injury and inflammation in mice subjected to permanent MCAO (Mota et al., 2020).

The beneficial effects of the combination of resveratrol and MS-275 are mediated by the reversion of the mismatch of RelA acetylation state by respectively reducing the acetylation at the K310 *via* SIRT1 activation and enhancing the RelA general acetylation (Lanzillotta et al., 2013). The drug combination also reverted the histone H3 deacetylation produced by the ischemic injury (Lanzillotta et al., 2013). Of note, the drug effect was sustained by the resveratrol-promoted AMPK activation that, by increasing generation of acetyl-CoA, can support HAT activity (Lanzillotta et al., 2013). Moreover, AMPK could also corroborate SIRT1 activation by resveratrol *via* induction of NAD+, the fundamental co-factor for class III HDACs (Ruderman et al., 2010).

The substitution of MS-275 with valproate (VPA), an antiepileptic/mood stabilizer endowed with inhibitory activity for class I and class IIa HDACs (Perucca, 2002; Gurvich et al., 2004), in association with resveratrol exerted synergistic neuroprotection in the OGD cellular model of brain ischemia, by correcting the pathological acetylation state of RelA and reverting the histone H3 deacetylation (Faggi et al., 2018). Moreover, a single intraperitoneal administration of the association of resveratrol and VPA synergistically reduced infarct volume and neurological deficits in the tMCAO mouse model of ischemic stroke (Faggi et al., 2018). Interestingly, VPA increased RelA general acetylation *in vivo* (Chen S et al., 2018).

Resveratrol and many HDAC inhibitors have been individually studied also against ALS (Chuang et al., 2009; Carrera-Juliá et al., 2020; Shukla and Tekwani, 2020; Klingl et al., 2021; Novak et al., 2021). The major effects of resveratrol and the main HDAC inhibitors tested individually or in combination in ALS preclinical models or patients are reported in Table 1 and described in the following sections.

**TABLE 1:** Effect of resveratrol and the main HDAC inhibitors administered individually or in combination in ALS animal models and patients.

**Drug**	**Treatment**	**Experimental model**	**Stage of study**	**Findings**	**Sexual dimorphism**	**References**
Resveratrol	10 μM, 3 h	NSC34-G93A cells, primary mouse MNs	preclinical	↑ cell viability	not evaluated	Barber et al. (2009)
				↓ oxidative stress		
	0.25 µM, 24 h	SOD1(G93A) primary rat cortical neurons	preclinical	↑ cell viability	not evaluated	Kim et al. (2007)
	10 μM, 24 h	hSOD1(G93A) human VSC4.1 MNs-like cells	preclinical	↑ cell viability	not evaluated	Wang et al. (2011)
				↓ apoptosis		
	1 μM, 12 h	BM-MSCs	preclinical	↑ neuronal differentiation, restoration of SIRT1/AMPK activities	not evaluated	Yun et al. (2019)
	0.3 µM, 24 h	rat cortical MNs treated with CSF from ALS patients	preclinical	↑ cell viability, restoration of Ca^2+^ homeostasis	not evaluated	Yáñez et al. (2011)
	160 mg/kg/day, oral	SOD1(G93A) mice, males and females	preclinical	↑ onset-time	↑ onset-time (15% males; 11% females)	Mancuso et al. (2014)
				↑ 9% survival		
				↓ MNs loss and microglia activation		
	25 mg/kg/day, oral	SOD1(G93A) mice, females	preclinical	no effect	not evaluated	Markert et al. (2010)
	20 mg/kg twice a week, i.p.	SOD1(G93A) mice, males	preclinical	↑ 10% onset-time	not evaluated	Han et al. (2012)
				↑ 7% survival		
				↓ MNs loss		
TSA	1 mg/kg/day, i.p.	SOD1(G93A) mice, males	preclinical	↑ 7% survival	not evaluated	Yoo and Ko, (2011)
				↓ MNs loss, gliosis, muscular atrophy and neuromuscular junction denervation		
NaPB	1 mM	NSC34-G93A cells	preclinical	↑ mitochondrial function	not evaluated	Li et al. (2022)
	400 mg/kg/day, i.p.	SOD1(G93A) mice, males	preclinical	↑ 21% survival	not evaluated	Ryu et al. (2005)
				↑ motor function and anti-apoptotic factors		
	400 mg/kg/day, i.p.	SOD1(G93A) mice	preclinical	↑ 13% survival	not evaluated	Petri et al. (2006)
				↑ motor function		
				↓ MNs loss		
	300 mg/kg/day, i.p.	SOD1(G93A) mice, males	preclinical	↑ 13% survival	not evaluated	Del Signore et al. (2009)
				↑ motor function		
	increasing dosage over 12 weeks to a maximum of 21 g/day, oral	ALS patients	phase II	safe and tolerable	not evaluated	Cudkowicz et al. (2009)
				no efficacy detected		
ACY-738	1 μM, overnight	ALS patients iPSC-derived MNs	preclinical	restoration of axonal transport	not evaluated	Guo et al. (2017)
	100 mg/kg, oral	Tg FUS^+/+^ mice, males and females	preclinical	↑ survival	↑ survival (76% males	Rossaert et al. (2019)
				= microglia and astroglia activation restoration of histone H3 acetylation	24% females)	
	100 mg/kg, oral	Tg FUS^+/+^ mice, males and females	preclinical	restoration of correct lipid metabolism	not evaluated	Burg et al. (2021)
Tubastatin A	1 μM, overnight	ALS patients iPSC-derived MNs	preclinical	restoration of axonal transport	not evaluated	Guo et al. (2017)
	1 μM, overnight	ALS patients iPSC-derived MNs	preclinical	restoration of axonal transport	not evaluated	Fazal et al. (2021)
MC1569	60 mg/kg/day, i.p.	SOD1(G93A) mice, males and females	preclinical	= survival	no sex-specific effect	Lapucci et al. (2017)
				↑ Glut uptake in spinal cord		
	40 mg/kg/day, i.p.	SOD1(G93A) mice, males and females	preclinical	↑ onset-time	no sex-specific effect	Buonvicino et al. (2018)
				= survival		
				= MNs loss		
				↑ muscle electrical potential		
				↑ expression of myogenic genes		
VPA	2 mM, 24 h	NSC34-G93A cells	preclinical	↑ cell viability, protection against H_2_O_2_ and Glut insults	not evaluated	Gyawali et al. (2022)
	0.6 mM, 72 h	NSC34-G93A cells	preclinical	↓ apoptosis	not evaluated	Jiang et al. (2016)
	10 μM, 48 h	rat primary MNs	preclinical	↑ cell viability	not evaluated	Ragancokova et al. (2010)
	500 mg/kg/day, oral	SOD1(G93A) mice, males	preclinical	= onset-time	not evaluated	Sugai et al. (2004)
				↑ 8% survival		
	300 mg/kg twice a day, i.p.	SOD1(G93A) mice, combined males and females	preclinical	↑ 8% onset-time	not evaluated	Feng et al. (2008)
				↑ 10% survival		
				↑ motor function		
	∼3 g/kg/day, oral	SOD1(G93A) mice, combined males and females	preclinical	= survival, ↓ MNs loss	not evaluated	Crochemore et al. (2009)
	250 mg/kg/day, i.p.	SOD1(G86R), male mice	preclinical	↑ 10% onset-time, = survival	not evaluated	Rouaux et al. (2007)
	1,500 mg daily, oral	ALS patients	phase II	no effect in survival	not evaluated	Piepers et al. (2006)
Resveratrol + MS-275	136 μg/kg/day (resveratrol), 4 μg/kg/day (MS-275), i.p.	SOD1(G93A) mice, combined males and females	preclinical	↑ 25% onset-time	not evaluated	Schiaffino et al. (2018)
				↑ 12% survival		
				↓ MNs loss		
				↑ anti-apoptotic and neurotrophic factors		
				= microglia activation		
				restoration of RelA		
				and histone H3 acetylation		
Resveratrol + VPA	136 μg/kg/day (resveratrol), 40 μg/kg/day (VPA), i.p.	SOD1(G93A) mice, males and females	preclinical	↑ 17% onset-time	↑ 27% onset-time in males, no significant effect in females; ↑ 6% survival in females, no significant effect in males	Bankole et al. (2022)
				↑ 7% survival		
				↓ MNs loss		
				↑ anti-apoptotic and neurotrophic factors		
				↓ microglia activation, restoration of RelA		
				and histone H3 acetylation		

If reported in the cited papers, *in vivo* results have been described by sex, and sexual dimorphism indicated. BM-MSCs: bone marrow-mesenchymal stem cells; CSF: cerebrospinal fluid; Glut: glutamate; i.p.: intraperitoneal; iPSC: induced pluripotent stem cell; MNs: motor neurons; NaPB: sodium phenylbutyrate; TSA: trichostatin A; VPA: valproate.

## Effect of resveratrol in ALS preclinical models and patients

A protective effect of resveratrol in ALS was demonstrated in neuronal cell lines expressing the SOD1(G93A) mutant (Kim et al., 2007; Barber et al., 2009; Wang et al., 2011). Interestingly, it has been reported that bone marrow mesenchymal stem cells from ALS patients displayed down-regulation of AMPK/SIRT1 signalling, which was rescued by treatment with resveratrol (Yun et al., 2019). In addition, resveratrol prevented the neurotoxic effect of cerebrospinal fluid (CSF) from ALS patients on cultured MNs (Yáñez et al., 2011).

Dietary treatment of SOD1(G93A) male and female mice with resveratrol (160 mg/kg/day) delayed disease onset, extended lifespan of approximately 10%, and preserved MNs survival (Mancuso et al., 2014). Interestingly, resveratrol delayed disease onset in a sexually dimorphic fashion, postponing symptoms onset of 2 weeks in males and 1 week in females (corresponding to a delay of approximately 15% and 11%, respectively).

Another study reported that dietary resveratrol at the dose 25 mg/kg/day did not promote functional effects in SOD1(G93A) female mice (Markert et al., 2010). Conversely, Han and colleagues reported that intraperitoneal administration of resveratrol at the dose 20 mg/kg twice a week delayed disease onset, extended survival of 7% and reduced MNs loss in SOD1(G93A) male mice (Han et al., 2012).

## Effect of HDAC inhibitors in ALS preclinical models and patients

Intraperitoneal treatment of SOD1(G93A) male mice with 1 mg/kg/day of trichostatin A (TSA), a pan-HDAC inhibitor of all zinc-dependent HDACs (Khan et al., 2008), promoted an increase of 7% in lifespan, together with a reduction of MNs loss, gliosis, muscular atrophy and neuromuscular junction denervation (Yoo and Ko, 2011). However, studies in human lymphoblasts *in vitro* pointed out genotoxic effects of TSA, raising doubts about a possible clinical use of the molecule (Olaharski et al., 2006).

Sodium phenylbutyrate (NaPB) is an inhibitor of HDAC class I and IIa (Kusaczuk et al., 2015). A recent study has shown the beneficial effect of NaPB in improving mitochondrial bioenergetics in a cellular model of ALS (Li et al., 2022). NaPB injected intraperitoneally at the dose of 400 mg/kg/day, prolonged the survival by 21%, ameliorated motor function and promoted expression of anti-apoptotic genes in SOD1(G93A) male mice (Ryu et al., 2005). The effect of NaPB on SOD1(G93A) mice was confirmed in another study, where the molecule injected intraperitoneally at the same dose significantly increased motor function, extended survival by 13% and attenuated MNs loss (Petri et al., 2006). In a third study, daily intraperitoneal treatment of SOD1(G93A) male mice with NaPB at the dose 300 mg/kg improved survival by approximately 13% while ameliorating body weight loss and grip strength (Del Signore et al., 2009). A phase II clinical trial studying the effect of an increasing dosage of NaPB over 12 weeks to a maximum of 21 g/day, reported the safety and tolerability of the drug, but did not evaluate its efficacy in ALS (Cudkowicz et al., 2009).

ACY-738 is a novel HDAC inhibitor selective for HDACs 1, 2, 3 and 6 (Mithraprabhu et al., 2013; Jochems et al., 2014). Treatment with ACY-738 rescued axonal transport deficits in MNs expressing FUS mutation derived from induced pluripotent stem cells (iPSCs) from ALS patients (Guo et al., 2017). Recently Van Den Bosch and colleagues have shown that the oral treatment of the ALS transgenic mouse model Tg FUS^+/+^ (both males and females) with ACY-738 (100 mg/kg) slowed down the disease progression, and improved the lifespan (Rossaert et al., 2019). Interestingly, the authors reported a larger survival extension in males (76% in males, 24% in females) (Rossaert et al., 2019). The beneficial effect of the molecule was associated with a mitigation of lipid metabolism alterations and a restoration of global histone acetylation, but not with a reduction of astrocytosis nor microgliosis (Rossaert et al., 2019; Burg et al., 2021).

Tubastatin A is a highly selective inhibitor of HDAC 6 (Butler et al., 2010) which has been investigated in different neurological disease animal models (Shen et al., 2020). Similarly to ACY-738, pharmacological inhibition of HDAC 6 by tubastatin A reverted axonal transport deficits in ALS patient-derived MNs with mutations for FUS and TDP-43 (Guo et al., 2017; Fazal et al., 2021).

MC1569 is a novel selective inhibitor of the HDAC class II (Mai et al., 2005). Treatment of SOD1(G93A) male and female mice with MC1568 (60 mg/kg/day, i.p.) restored glutamate uptake capacity in spinal cord, but did not increase lifespan (Lapucci et al., 2017). In a second study, i.p. treatment of SOD1(G93A) male and female mice with the drug at 40 mg/kg/day promoted early improvement of motor performances that disappeared at later stages of disease (Buonvicino et al., 2018). The transient motor improvement was coupled with increased skeletal muscle electrical potentials and muscle expression of myogenic genes, but not with a protection of MNs from neurodegeneration (Buonvicino et al., 2018). No evidence of sex-specific effect was found.

VPA treatment reduced neurotoxicity in motor neuron cellular models of ALS (Ragancokova et al., 2010; Jiang et al., 2016; Gyawali et al., 2022). *In vivo*, oral administration of VPA at the antiepileptic dose of approximately 500 mg/kg/day increased lifespan by 8% without delaying the disease onset in SOD1(G93A) male mice (Sugai et al., 2004). Feng and colleagues reported that VPA treatment (300 mg/kg twice a day, i.p.) in SOD1(G93A) male and female mice delayed motor deficits onset by 8%, improved lifespan by 10%, and had beneficial effects on motor dysfunction (Feng et al., 2008). In another study, oral treatment of SOD1(G93A) male and female mice with the drug at antiepileptic dose slowed down MNs loss without significantly improving lifespan (Crochemore et al., 2009). VPA, when administered intraperitoneally at the antiepileptic dose of 250 mg/kg/day to SOD1(G86R) male mice, delayed the disease onset of 10%, but failed in improving mean survival (Rouaux et al., 2007). VPA efficacy has been investigated also in phase II clinical trials for ALS, but VPA-treated subjects (1,500 mg daily) did not show a difference in survival or disease progression rate compared to placebo-treated patients (Piepers et al., 2006).

## The association of resveratrol and HDAC inhibitors in the ALS treatment

It has been shown the resveratrol and HDAC inhibitors at very low doses can synergize in promoting neuroprotection in the SOD1(G93A) mouse model of ALS (Schiaffino et al., 2018; Bankole et al., 2022).

When administered to combined male and female SOD1(G93A) mice, the association resveratrol (136 μg/kg/day) and MS-275 (4 μg/kg/day) delayed symptoms’ onset by 3 weeks and prolonged lifespan by 2 weeks, corresponding to an increase of 25% and 12%, respectively (Schiaffino et al., 2018). Furthermore, the treatment rescued MNs, and increased the levels of anti-apoptotic B-cell lymphoma-extra large (Bcl-xL) and neurotrophic Brain-Derived Neurotrophic Factor (BDNF) in the lumbar spinal cord, without modifying microglia activation (Schiaffino et al., 2018).

In a similar fashion, the treatment of SOD1(G93A) male and female mice with the association resveratrol (136 μg/kg/day) and VPA (40 μg/kg/day) promoted a significant improvement in motor performances, the delay of disease onset, and longer survival (Bankole et al., 2022). Moreover, the epigenetic drugs protected MNs from neurodegeneration, reduced immunoreactivity of microglia, and increased expression of Bcl-xL and BDNF levels in the lumbar spinal cord (Bankole et al., 2022).

In accordance with studies on brain ischemia models (Lanzillotta et al., 2013; Faggi et al., 2018), the beneficial effects promoted by the association of resveratrol and HDAC inhibitors was coupled to the rescue of RelA and the histone 3 acetylation state, and of AMPK activation (Schiaffino et al., 2018; Bankole et al., 2022), indicating a mechanism of action based on the reversion of the mismatch of RelA and histone acetylation also in ALS.

It is important to note that resveratrol can also modify the acetylation status of other proteins potentially involved in ALS pathogenesis. For example, resveratrol was able to deacetylate p53 and the peroxisome proliferator-activated receptor gamma coactivator1alpha (PGC1-α) in preclinical models of ALS (Kim et al., 2007; Mancuso et al., 2014). Both the proteins have been involved in mechanisms of MNs death (Ranganathan and Bowser, 2010; Lazo-Gómez et al., 2013), and their deacetylation has been associated with neuroprotection (Hasegawa and Yoshikawa, 2008; Panes et al., 2022). Therefore, it can be speculated that other mechanisms, besides the modulation of RelA and histone acetylation, may support the beneficial action of this pharmacological association.

**FIGURE 1:**
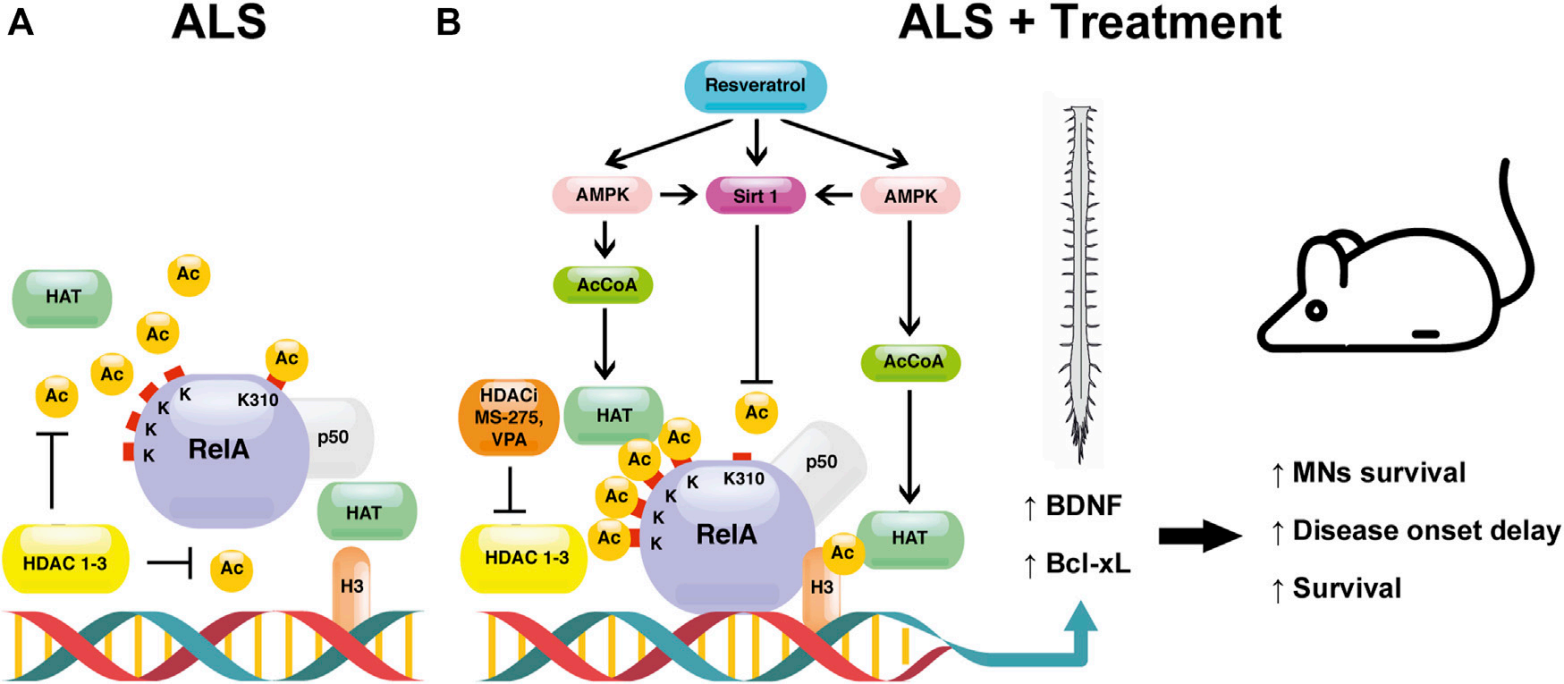
The proposed mechanism underlying the effect of the combination between resveratrol and HDAC inhibitors (HDACi) [MS-275 or valproate (VPA)] in ALS. **(A)** In the spinal cord of the SOD1(G93A) ALS mouse model, NF-κB/RelA is present in its aberrantly acetylated form, consisting of a hypoacetylated state with the exception of the residue K310 (ALS). In this pathological condition, the acetylation of histone H3 is decreased. **(B)** The treatment of SOD1(G93A) mice with the combination resveratrol/HDAC inhibitors reverts the aberrant RelA acetylation (ALS + treatment). HDAC inhibitors, by blocking HDAC activity, induce HAT-mediated acetylation of both RelA and H3 histone. Resveratrol activates the class III NAD+ -dependent HDAC SIRT1, promoting the deacetylation of RelA at residue K310. Moreover, resveratrol stimulates AMPK pathway, leading to an increase of NAD+ and AcCoA levels, enhancing the activation of SIRT1 and HATs, respectively. The modulation of RelA and H3 acetylation in the nucleus promotes the transcription of the anti-apoptotic Bcl-xL and the neurotrophic BDNF factors. Altogether, the treatment leads to a protection of MNs, a delay of symptoms’ onset, and longer survival.

Figure 1 depicts the mechanisms responsible for NF-κB/RelA and H3 histone acetylation upon treatment with combination of resveratrol and HDAC inhibitors in ALS mice.

The SOD1(G93A) mouse model displayed a sexually dimorphic behavior in response to the association of resveratrol and VPA (Bankole et al., 2022). Specifically, in accordance with a previous study investigating the effect of resveratrol in SOD1(G93A) mice (Mancuso et al., 2014), males showed positive outcomes in the early phases of the disease (onset delay of 27%). Conversely, only in females the epigenetic drugs reduced motor deficits in a later phase of the disease and prolonged survival by 6%. These findings suggest a possible action of resveratrol and VPA on specific sex-related molecular targets. For example, it is plausible that these compounds could potentiate the neuroprotective effect of female sex steroids *in vivo* (Bankole et al., 2022). In support of this, both resveratrol and VPA are endowed with estrogenic properties (Stempin et al., 2013; Qasem, 2020).

## Conclusion

In conclusions, recent evidence supports that the pathological RelA acetylation and histone hypoacetylation may represent an appealing pharmacological target for ALS treatment. The correction of pathological acetylation state in the SOD1(G93A) ALS model has been achieved by the synergistic combination of resveratrol with the class I HDAC inhibitors MS-275 and VPA (Schiaffino et al., 2018; Bankole et al., 2022).

The doses of the epigenetic drugs active in combination were extremely low, in contrast to those reported in ALS studies employing individual molecules, where the modulation of the enzymatic activity of HDAC requires a very high concentration. Administration of individual molecules promoted a delay of disease onset and an extension of lifespan sometimes comparable, or even better than that achieved by the association resveratrol and MS-275 or VPA (Schiaffino et al., 2018; Bankole et al., 2022), but at doses several folds higher. In some cases the beneficial outcomes promoted by individual-molecule treatment were overshadowed by severe side effects.

The low drug doses used in the association could minimize possible side or off-target effects and modulate better the neuroprotective action. For example, preclinical and clinical studies investigating the use of VPA in ALS treatment failed to provide real effective results. If VPA protected MNs likely *via* inhibition of class I HDACs, the drug did not avoid denervation of neuromuscular junction at late stages, possibly because of concomitant inhibition of class IIa HDAC 4 at the high doses used (Boutillier et al., 2019; Pigna et al., 2019). The fact that VPA and resveratrol impinge on different molecular targets (class I HDACs, SIRT1 and AMPK), and their low doses, could reduce off-target effects and lead to better outcomes.

In light of these considerations, we advocate further research on different ALS models and clinical trials aimed to investigate the synergistic effect and the mechanism of action of the combination of resveratrol with various HDAC inhibitors. It is worth noting that, in an effort to target different ALS mechanisms, some of the described HDAC inhibitors have been already successfully tested in combination with other molecules, including riluzole (Del Signore et al., 2009), the catalytic antioxidant AEOL 10150 (Petri et al., 2006), and the mood stabilizer lithium (Feng et al., 2008). Therefore, the combination of resveratrol with HDAC inhibitors could be also tested with the additional compounds above mentioned, and potentially others.

Finally, future studies focusing on the effects of these compounds on sex-related molecular targets will be necessary to define sex-specific treatment strategies aimed to improve therapeutic options for ALS patients.
